# The value of three narrow-band imaging model in the diagnosis of small colorectal polyps

**DOI:** 10.1038/s41598-020-78708-1

**Published:** 2020-12-16

**Authors:** Jie Sha, Pin Wang, Nan Sang, Huiai Zhang, Aiping Yang, Lei Chen, Zewei Gong, Chao Li, Yumeng Qin, Xueliang Li, Yong Ji, Feng Gao

**Affiliations:** 1 Department of Gastroenterology, Jingjiang People’s Hospital, Jingjiang, 214500 Jiangsu China; 2 Department of Gastroenterology, Nanjing Drum Tower Hospital, Affiliated Hospital of Nanjing University Medical School, Nanjing, 210008 Jiangsu China; 3 Department of Gastroenterology, The Geriatric Hospital of Nanjing Medical University, Nanjing, 210024 Jiangsu China; 4 Department of Gastroenterology, Taixing People’s Hospital, Taizhou, 225400 Jiangsu China; 5 Department of Gastroenterology, The First Affiliated Hospital of Nanjing Medical University, Nanjing, 210029 Jiangsu China

**Keywords:** Colon cancer, Cancer prevention

## Abstract

This prospective study was aimed to evaluate the clinical value of narrow-band imaging (NBI), magnification endoscopy with narrow-band imaging (NBIME) and magnification endoscopy with acetic acid enhancement and narrow-band imaging (AA-NBIME) in the diagnosis of small colorectal polyps. We studied 261 small colorectal polyps from 122 patients with the use of above three techniques. Lesions were resected for histopathological analysis. The endoscopic images were independently reviewed by three experts and three non-experts and the diagnostic accuracy and image definition were compared among the modalities. The “experts-agreed” diagnostic accuracy was 87.7% for NBI versus 91.6% for NBIME versus 94.6% for AA-NBIME. The “non-experts-agreed” diagnostic accuracy was 80.1% for NBI versus 84.3% for NBIME versus 89.3% for AA-NBIME. All experts and non-experts diagnosed the small colorectal polyps statistically more accurately with AA-NBIME than NBI (*P* < 0.05). In all three modalities, the expert group’s diagnostic accuracies were statistically significantly higher compared with the non-expert group. For experts, the Kappa values for AA-NBIME, NBIME and NBI diagnosis were 0.962 (0.892–1.032), 0.577 (0.507–0.647) and 0.567 (0.497–0.637), respectively; while for nonexperts, 0.818 (0.748–0.888), 0.532 (0.462–0.602) and 0.530 (0.460–0.600). This demonstrated a good reproducibility of AA-NBIME diagnosis. The average scores (experts and non-experts) of images acquired using AA-NBIME were significantly higher than those acquired using NBIME and ME (*P* < 0.05). AA-NBIME is a promising tool to clearly visualize the mucosal pit pattern (PP) of colorectal polyps for better differentiating neoplastic polyps from non-neoplastic ones.

## Introduction

Colorectal cancer (CRC) is the third most common cancer and the fourth leading cause of cancer death in the world^1^. Nowadays it is generally accepted that adenomatous polyps (AP) are precursors of CRC^2^, and their removal significantly reduces the incidence of CRC^3,4^. However, the conventional colonoscopy is not enough to distinguish non-neoplastic from neoplastic polyps. Hence, a considerable proportion of polyps are proved to be non-neoplastic according to histopathological assessment and they are unnecessary to be resected and sent for histopathological analysis, which may add to the potential cost and anxiety of patients due to the delay of pathology results. Differentiating neoplastic from non-neoplastic polyps in a more effective way is essential for determining appropriate treatment.

Much effort has been made on the new technologies to improve diagnostic accuracy to differentiate neoplastic from non-neoplastic colorectal lesions. NBI is a technology that uses 3 optical filters for red-blue-green sequential illumination and narrows the bandwidth of the spectral transmittance supporting the imaging of mucosal pit patterns (PPs) and capillary vessels^5^. In 2012, NBI International Colorectal Endoscopic (NICE) classification using NBI was developed and also validated internationally applicable in diagnosing colorectal polyps^6^. Magnification endoscopy combined with NBI (NBIME) has been reported to better show the details of mucosal PPs and capillaries; therefore, this technique has become useful to distinguish neoplastic from non-neoplastic lesions according to the NICE classification. However, in some cases, it is hard to interpret the findings of NBIME^7,8^, and is not known as to the potential advantage of NBIME over NBI in the histological differential diagnosis of Small colorectal polyps.

Magnification endoscopy with acetic acid-enhanced NBI (AA-NBIME) is considered to be an effective method for observing the microstructure of the mucosal surface^9^. This modality can vividly observe the deep brown glandular epithelial crypts and the whitish area between the crypts. This whitish area is a reversible molecular structure change of cellular proteins induced by acetic acid. The duration can be from a few seconds to a few minutes^10^. In patients with colorectal tumors, AA-NBIME enabled a clear visualization of the pit patterns (the shape of the opening of a glandular crypt) and prediction of the histologic features, which technically simplifies the surgical procedure and thus reduces the time^11^. Unfortunately, there are only few published articles concerned with the comparison among NBI, NBIME and AA-NBIME on the diagnosis of small colorectal polyps. In this study, we aimed to compare the diagnostic accuracy among NBI, NBIME and AA-NBIME.

## Results

### Clinical characteristics of participants

A total of 261 polyps were identified and analyzed in 122 patients. 2 polyps were excluded due to insufficient enhancement by acetic acid. 85 were men and 37 were women. The mean age of all patients with polyps was 54.2 years (range 43.4–65.0 years). The size of 132 polyps was in 1–5 mm and 129 in 6–10 mm. 79 polyps were found in the location of right colon (cecum, ascending and transverse colon), 119 in the left colon (descending and sigmoid colon) and 63 in the rectum (Table 1).

**Table 1:** Clinical characteristics.

Patients (n)	122
Age (years) mean ± SD	54.2 ± 11.8
Male	85
Female	37
Polyps (n)	261
1–5 mm	132
6–10 mm	129
Right colon	79
Left colon	119
Rectum	63

### Comparison between observations and histologic diagnosis under different modalities

Predicted and actual histology for small colorectal polyps were given as follows. For three experts, the number of colorectal tumorous lesions diagnosed by NBI, NBIME and AA-NBIME were 163, 161 and 161, among which 148, 152 and 156 were confirmed by histopathologic assessment. The number of non-neoplastic polyps diagnosed by NBI, NBIME and AA-NBIME were 98, 100 and 100, among which 81, 87 and 91 were confirmed by histopathologic assessment. For three non-experts, the number of colorectal tumorous lesions diagnosed by NBI, NBIME and AA-NBIME were 157, 158 and 165, among which 135, 141 and 151 were confirmed by histopathologic assessment. The number of non-neoplastic polyps diagnosed by NBI, NBIME and AA-NBIME were 104, 103 and 96, among which 74, 79 and 82 were confirmed by histopathologic assessment (Table 2).

**Table 2:** Correlation between (a) “experts-agreed diagnosis”, (b) “non-experts-agreed diagnosis” for each modality and histopathologic type.

	Neoplastic, n (%)	Non-neoplastic, n (%)
**(a) Experts-agreed diagnosis**		
NBI		
Neoplastic	148 (56.7)	15 (5.7)
Non-neoplastic	17 (6.5)	81 (31.0)
NBIME		
Neoplastic	152 (58.2)	9 (3.4)
Non-neoplastic	13 (5.0)	87 (33.3)
AA-NBIME		
Neoplastic	156 (60.0)	5 (2.0)
Non-neoplastic	9 (3.4)	91 (34.9)
**(b) Non-experts-agreed diagnosis**		
NBI		
Neoplastic	135 (51.7)	22 (8.4)
Non-neoplastic	30 (11.5)	74 (28.4)
NBIME		
Neoplastic	141 (54.0)	17 (6.5)
Non-neoplastic	24 (9.2)	79 (30.3)
AA-NBIME		
Neoplastic	151 (57.9)	14 (5.4)
Non-neoplastic	14 (5.4)	82 (31.4)

#### Diagnostic accuracy of small colorectal polyps in the expert group

For three experts, as shown in Table 3, AA-NBIME exhibited a significantly higher specificity, positive predictive value (PPV), negative predictive value (NPV) and accuracy than those of NBI (*P* < 0.05). The diagnostic sensitivity, specificity, negative predictive value (NPV) and accuracy of AA-NBIME were all higher than NBIME. However, no statistically significant differences were found between AA-NBIME and NBIME. Moreover, for NBIME, the diagnostic sensitivity, specificity, positive predictive value (PPV), negative predictive values (NPV) were all significantly higher than those for NBI (*P* < 0.05). The accuracy was also higher than NBI. However, this was only a trend without statistically significant difference between NBIME and NBI (Table 3).

**Table 3:** Diagnostic performance of optical diagnosis of colorectal neoplastic polyps by three modalities for the expert group.

Modality	NBI (95% CI)	NBIME (95% CI )	AA-NBIME (95% CI)	$P_{{NBI vs AA{ - }NBIME}}$	$P_{NBI vs NBIME }$	$P_{{NBIME vs AA{ - }NBIME}}$
Sensitivity	89.7% (85.1–94.3)	92.1% (88.0–96.2)	94.5% (91.1–98.0)	0.117	0.045	0.394
Specificity	84.4% (77.1–91.6)	90.6% (84.8–96.5)	94.8% (90.3–99.2)	0.008	0.014	0.285
Accuracy	87.7% (83.2–91.2)	91.6% (87.5–94.4)	94.6% (91.1–96.9)	0.009	0.196	0.227
PPV	90.8% (86.4–95.2)	94.4% (90.9–98.0)	96.9% (94.2–99.6)	0.006	0.011	0.266
NPV	82.7% (75.2–90.1)	87.0% (80.4–93.6)	91.0% (85.4–96.6)	0.069	0.017	0.342

*PPV* positive predictive value, *NPV* negative predictive value.

#### Diagnostic accuracy of small colorectal polyps in the non-expert group

For three non-experts, AA-NBIME had a significantly higher sensitivity, positive predictive value (PPV), negative predictive value (NPV) and accuracy than NBI (*P* < 0.05). AA-NBIME exhibited a significantly higher sensitivity and negative predictive value (NPV) than those of NBIME (*P* < 0.05). The diagnostic specificity, positive predictive value (PPV) and accuracy of AA-NBIME were all higher than NBIME. However, no statistically significant differences were found between them. What’s more, the diagnostic sensitivity, specificity, positive predictive value (PPV) and negative predictive value (NPV) of NBIME were all significantly higher than NBI (*P* < 0.05). The accuracy was also higher than NBI. However, this was only a trend without statistically significant difference between NBIME and NBI (Table 4).

**Table 4:** Diagnostic performance of optical diagnosis of colorectal neoplastic polyps by three modalities for the non-expert group.

Modality	NBI (95% CI)	NBIME (95% CI )	AA-NBIME (95% CI)	$P_{{NBI vs AA{ - }NBIME}}$	$P_{NBI vs NBIME}$	$P_{{NBIME vs AA{ - }NBIME}}$
Sensitivity	81.8% (75.9–87.7)	85.5% (80.1–90.8)	91.5% (87.3–95.8)	< 0.001	0.014	0.033
Specificity	77.1% (68.7–85.5)	82.3% (74.7–89.9)	85.4% (78.4–92.5)	0.157	0.025	0.590
Accuracy	80.1% (74.8–84.5)	84.3% (79.4–88.2)	89.3% (84.9–92.5)	0.005	0.253	0.121
PPV	86.0% (80.6–91.4)	89.2% (84.4–94.1)	91.5% (87.3–95.8)	0.083	0.012	0.469
NPV	71.2% (62.4–79.9)	76.7% (68.5–84.9)	85.4% (78.4–92.5)	< 0.001	0.004	0.029

*PPV* positive predictive value, *NPV* negative predictive value.

### Diagnostic accuracy of small colorectal polyps between expert group and non-expert group among different modalities

In all three modalities, the non-expert group’s diagnostic accuracies were statistically significantly lower compared with the expert group (NBI, *P* = 0.024; NBIME, *P* = 0.011; AA-NBIME, *P* = 0.024) (Table 5).

**Table 5:** Diagnostic accuracy of small colorectal polyps between the expert group and non-expert group for different modalities.

Modality	Accuracy		*P*
	Expert group (%)	Non-expert group (%)	
NBI	87.7	80.1	0.024
NBIME	91.6	84.3	0.011
AA-NBIME	94.6	89.3	0.024

### Comparison of the interobserver diagnostic agreement among ME, NBIME, and AA-NBIME

For the expert group, the kappa values, expressed as 95% confidence interval (CI), for AA-NBIME, NBIME and ME were 0.962 (0.892–1.032), 0.577 (0.507–0.647), 0.567 (0.497–0.637), respectively, showing “almost perfect” agreement for AA-NBIME, “substantial” agreement for NBIME, and “moderate” agreement for ME. For the non-expert group, the kappa values (95%CI) for AA-NBIME, NBIME and ME were 0.818 (0.748–0.888), 0.532 (0.462–0.602) and 0.530 (0.460–0.600), respectively, showing almost a perfect agreement for AA-NBIME, a moderate agreement not only for NBIME but also for ME (Table 6).

**Table 6:** Kappa statistics of interobserver agreement for diagnosis among three modalities for experts and non-experts.

Modality	Kappa value (95% CI)	
	Expert group	Non-expert group
ME	0.567 (0.497–0.637)	0.530 (0.460–0.600)
NBIME	0.577 (0.507–0.647)	0.532 (0.462–0.602)
AA-NBIME	0.962 (0.892–1.032)	0.818 (0.748–0.888)

### Comparison of the image quality among NBI, NBIME, and AA-NBIME

With higher values indicating better quality, the scores were: For expert group, NBI, 2.69 ± 0.85, NBIME, 2.96 ± 0.77, AA-NBIME, 3.20 ± 0.66. For non-expert group, NBI, 2.32 ± 1.15, NBIME, 3.23 ± 0.89, AA-NBIME, 3.39 ± 0.71. The average scores (experts and non-experts) of images acquired using AA-NBIME and NBIME were significantly higher than those acquired using NBI (*P* < 0.001).The average scores (experts and non-experts) of images acquired using AA-NBIME were significantly higher than those acquired using NBIME (*P* < 0.001) (Table 7).

**Table 7:** Comparison of the image quality among NBI, NBIME, and AA-NBIME.

Modality	Image quality scores (*t* ± *s*)	
	Expert group	Non-expert group
NBI	2.69 ± 0.85***	2.32 ± 1.15***
NBIME	2.96 ± 0.77***	3.23 ± 0.89***
AA-NBIME	3.20 ± 0.66	3.39 ± 0.71

***AA-NBIME, NBIME versus NBI *P* < 0.001, ***AA-NBIME versus NBIME *P* < 0.001.

## Discussion

It is widely accepted that most colorectal carcinomas appear to arise from adenomas^2^, and the removal of adenomatous polyps by colonoscope has already result in significant reductions in the incidence of CRC^3^. It is generally agreed that regular colonoscopy surveillance is necessary after these polypectomies. It has been reported that more than 90% of colonoscopy-identified polyps were small polyps (6–9 mm) or diminutive polyps (< 5 mm), and diminutive polyps are dominant^12,13^. The majority of small colon polyps are non-neoplastic, and most of which are hyperplastic in nature^12,13^. Therefore, these polypectomies are unnecessary to perform, posing risks of bleeding and perforation^14,15^. Real-time optical diagnosis of recto-sigmoid small polyps would allow hyperplastic polyps to be left in situ and adenomatous polyps to be removed without histopathology^16^.

NBI is a technology that uses 3 optical filters for red-blue-green sequential illumination and narrows the bandwidth of the spectral transmittance supporting the imaging of mucosal PPs and capillary vessels^5^. NBI can be switched on and off with a button on the endoscope and save the additional cost for dye spraying. NBI in combination with magnifying endoscopy is a promising tool for superior detection of mucosal PP and microvasculature details^10,17–20^, in order to differentiate non-neoplastic from neoplastic colorectal polyps^5^. Iwatate et al.^21^ reported that magnifying endoscopy could improve the performance of NBI in distinguishing neoplastic from non-neoplastic colorectal lesions according to NICE classification. We did not observe any significant benefit of NBIME over NBI for improving the diagnostic accuracy of small colorectal polyps; however, diagnostic accuracy and image quality of NBIME were better than those of NBI.

Acetic acid is a hydrophilic organic acid. It can infiltrate the crypt easily by its small molecular weight, quickly discolor the intervening part between crypts and the marginal crypt epithelium to enable the good visualization of pits^11^. It was reported that magnification endoscopy with acetic acid enhancement and narrow-band imaging is useful for visualizing mucosal microstructure patterns of colorectal polyps.

Magnification endoscopy with crystal violet staining enables clear visualization of the PPs of the colorectal polyps, and helps to differentiate neoplastic from non-neoplastic lesions^22–24^. Kudo et al.^25^ classified different morphological features into six PPs based on this techque (Type I, Type II, Type IIIS, Type IIIL, Type IV, Type V), with types I and II regarded as non-polyps, types III (including IIIL and IIIs), IV considered to be adenoma and type V classified as adenocarcinoma^26^. Despite having the merit of effectively evaluating colorectal polyps, this technique requires the use of 0.05% crystal violet spraying and is technically hard for endoscopists. It’s reported that AA-NBIME diagnosis was not inferior that of magnification endoscopy with crystal violet staining in the diagnostic accuracy of colorectal polyps. In addition, it is easier to operate^27^. This study finds that for experts and non-experts, the diagnostic accuracy of AA-NBIME were all significantly higher than NBI. AA-NBIME didn’t show a statistically significantly higher diagnostic accuracy compared to NBIME, however, the performance of AA-NBIME was better than that of NBIME in terms of diagnostic accuracy.

We also believe that, of all three modalities, the performance of expert group was better than that of the non-expert group in terms of diagnostic accuracy. Rogart et al.^24^ reported that diagnostic accuracy using NBI significantly improved with the increasing experience level of endoscopists. They also pointed out that the experience from diagnosing nearly 130 polyps is essential for basic competency. This finding emphasized the importance of practice and experience of endoscopists, which agreed with our results.

In addition, acetic acid removes the mucus adherent to the colorectal polyps through breaking the disulfide bonds of mucus, enabling the better visualization of the lesions. In this study, the average scores (experts and non-experts) of images acquired using AA-NBIME were significantly higher than those acquired using NBIME and NBI.

AA-NBIME is a promising tool to clearly visualize the mucosal PP of colorectal polyps for better differentiating neoplastic polyps from non-neoplastic ones. In conclusion, for experts and non-experts, AA-NBIME showed statistically significantly higher diagnostic accuracy for small colorectal polyps.

There were also some limitations in this study. This study was performed at only two centers. Adequate evaluation of the efficacy of AA-NBIME needs a multicenter trial with more patients. What’s more, to maintain the image quality, all the endoscopic images were taken by a single experienced endoscopist. The procedure of magnification endoscopic is technically hard to non-experts, which might limit the applicability of AA-NBIME. We also acknowledge that although we distributed all the images in a random order intermixed with other lesions to the six endoscopists, the possibility that one endoscopist could simultaneously recognize the three modalities of the same lesion cannot be ruled out, which would interfere with his diagnosis of the lesion.

## Methods

### Participants

Patients scheduled for performing polypectomies (< 1 cm) and agreed with ME at Jiangsu Province Hospital and Jingjiang People’s Hospital between May 2017 and January 2018 were consecutively enrolled in this study. We excluded patients from enrolment who had coagulopathy or a platelet count less than 50,000/mm^3^ or lesions covered with adherent mucus or blood, insufficiently enhanced by the acetic acid. The study was performed in accordance with the Declaration of Helsinki and was approved by the Ethics Committee of the Jingjiang People’s Hospital, Jingjiang, Jiangsu, China and Jiangsu Province Hospital, Nanjing, Jiangsu, China. All patients signed written informed consent for every procedure.

### Endoscopic procedure and therapy

1. Bowel preparation: All patients were prepared for colonoscopy with 2L of polyethylene glycol-electrolyte solution and dimethicone administered on the morning of the examination.
2. Endoscopic Procedure: A conventional colonoscope was used first for routine colonoscopy. When the colonoscope arrived to ileocecus, reinspect the colon during withdrawal from the cecum. Once a polyp was detected, surface mucus was washed away with lukewarm water, and optical diagnosis was made using magnifying Olympus CF-H260Z colonoscopes. Endoscopic images were taken in the following order: Narrow-band imaging (NBI), Magnification endoscopy with narrow-band imaging (NBIME) and Magnification endoscopy with acetic acid (1.5%)-enhanced NBI (AA-NBIME). For AA-NBIME, 5–10 ml of 1.5% acetic acid solution was dripped onto the polyps using a special tube, which were inserted from the forceps channel of the endoscope and the microstructure of the same areas that had been observed by NBI and NBIME were photographed with NBIME under acetic acid-enhanced conditions. Diagnosis was made using NBIME under the enhancement of acetic acid. Endoscopic images were taken before and 15–30 s after spraying under the condition of full air inflation^28^. All endoscopic images were taken by a single expert endoscopist (Sha) under different modalities, who had experience of over 2000 cases of magnification colonoscopy with NBI.
3. Therapy: All polyps were removed by biopsy forceps or Endoscopic Mucosal Resection (EMR) and sent for histological assessment.

### Assessment of endoscopic images

According to NICE and Kudo PP classifications, all of the endoscopic images of NBI, NBIME and AA-NBIME were displayed for each modality in a random order, and NBIME and AA-NBIME images were displayed alone, without the risk of bias caused by corresponding NBI image.

**Figure 1:**
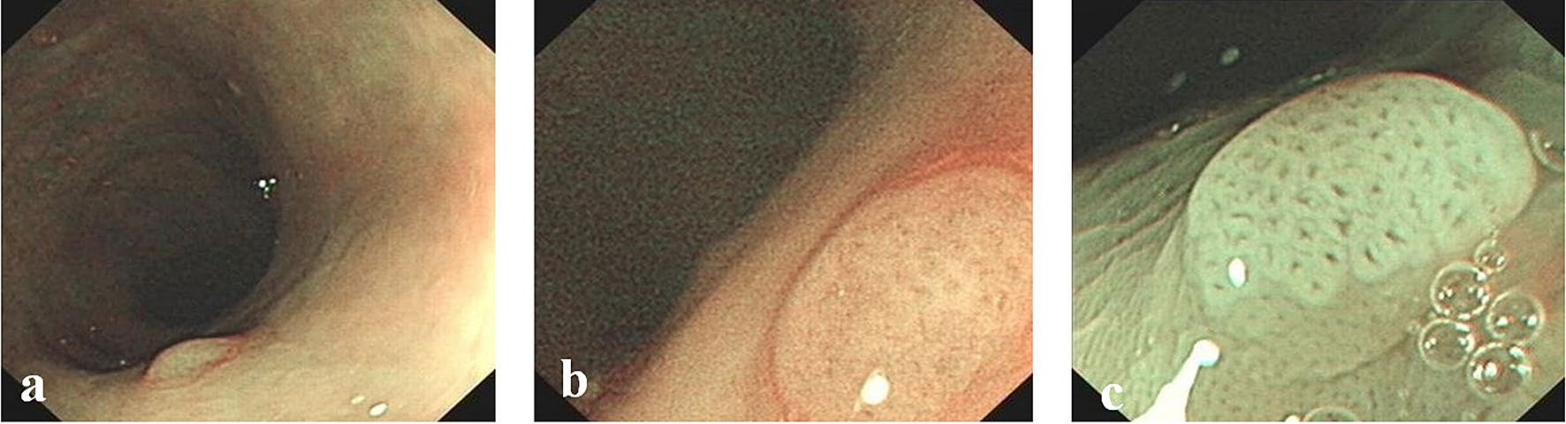
Surface patterns of non-neoplastic polyps viewed by different modalities: (**a**) NBI; (**b**) NBIME; (**c**) AA-NBIME.

**Figure 2:**
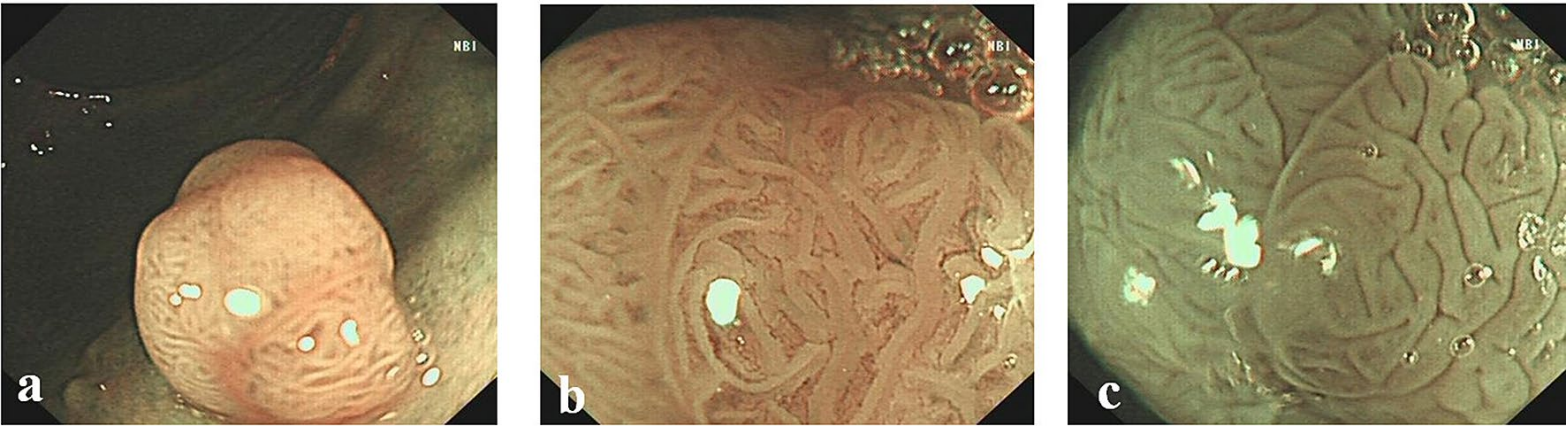
Surface patterns of neoplastic polyps viewed by different modalities: (**a**) NBI; (**b**) NBIME; (**c**) AA-NBIME.

The endoscopic images of each modality were independently reviewed by six endoscopists (three experts and three non-experts), who were all blinded to the final histological diagnosis. The three experts were well versed and had 5 years’ experience in narrow-band imaging and magnifying endoscopy in the colorectum and the three non-experts were proficient in conventional colonoscopy, while without any experience in narrow-band imaging or magnifying endoscopy. Images taken under NBI and NBIME were assessed according to NICE classification, while those taken under AA-NBIME were assessed according to Kudo PP classification. Diagnostic accuracy was compared among three modalities based on Histopathological results. Before starting the study, the non-expert group completed a training session on NICE and Kudo PP classifications. In both groups, the accuracy of the modality was included in the study only when two or three members had the same idea on the classification of the lesion. The NBI International Colorectal Endoscopic (NICE) classification was as follows: Type 1: Color: Same or lighter than background; Vessels: None, or isolated lacy vessels coursing across the lesion; Surface pattern: Dark or white spots of uniform size, or homogeneous absence of pattern (Fig. 1); Type 2: Color: Browner relative to background (verify color arises from vessels); Vessels: Brown vessels surrounding white structures; Surface pattern: Oval, tubular, or branched white structures surrounded by brown vessels (Fig. 2). Type 1 was most likely to be hyperplastic, while Type 2 was most likely to be adenoma^6^. The Kudo PP classification was as follows: Type I: round pits ; Type II: stellar or papillary pits ; Type III L: large tubular or roundish pits ; Type III S: small tubular or roundish pits ; Type IV: branch-like or gyrus-like pits ; Type V: non-structural pits^11^. Types I and II were classified as non − neoplastic, as shown in Fig. 1, whereas types III, IV and V were regarded as neoplastic lesions, as shown in Fig. 2. Image quality classification was as follows: score 1: unobservable; Score 2: blurred; score 3: less clear; score 4: clear.

To verify the clinical feasibility and availability of each diagnostic modality, kappa statistics were applied for analyzing an interobserver diagnostic agreement among each modality for the expert and non-expert groups.

### Histopathological assessment

All polyps were immediately fixed in 10% formalin and then standard H&E staining was performed. A single expert pathologist, who was blinded to the endoscopic assessment, judged the histologic characteristics of the specimens.

### Statistical analysis

The statistical analysis was performed using the SPSS for Windows Version 16.0 statistical software package. Comparisons for sensitivity, specificity, and accuracy among the three modalities were tested by paired Chi-square method. The differences in positive predictive value and negative predictive value of the three modalities were compared by the method proposed by Kosinski^29^. The image definition score is shown as x ± s and one-way analysis of variance was applied in order to compare the scores among three modalities. *P *value of < 0.05 was considered to be statistically significant.

With kappa statistics, we also analyzed the interobserver diagnostic agreement for the expert and non-expert groups. Data are expressed as point estimates of kappa with 95% CI. In theory, perfect disagreement has a kappa value of − 1.0, and a kappa value of  1.0 is considered perfect agreement. A value of 0 indicates an agreement by chance alone. According to the Landis and Koch scale, kappa values are graded as follows: 0.01–0.2 slight, 0.21–0.4 fair, 0.41–0.6 moderate, 0.61–0.8 substantial, and 0.81–1.0 almost perfect^30,31^.
